# Nucleotide Excision Repair and Vitamin D—Relevance for Skin Cancer Therapy

**DOI:** 10.3390/ijms17040372

**Published:** 2016-04-06

**Authors:** Elzbieta Pawlowska, Daniel Wysokinski, Janusz Blasiak

**Affiliations:** 1 Department of Orthodontics, Medical University of Lodz, 92-216 Lodz, Poland; elzbieta.pawlowska@umed.lodz.pl; 2 Department of Molecular Genetics, University of Lodz, 90-236 Lodz, Poland; dwysokinski@biol.uni.lodz.pl

**Keywords:** DNA damage, UV radiation, DNA repair, nucleotide excision repair, skin cancer, melanoma

## Abstract

Ultraviolet (UV) radiation is involved in almost all skin cancer cases, but on the other hand, it stimulates the production of pre-vitamin D3, whose active metabolite, 1,25-dihydroxyvitamin D3 (1,25VD3), plays important physiological functions on binding with its receptor (vitamin D receptor, VDR). UV-induced DNA damages in the form of cyclobutane pyrimidine dimers or (6-4)-pyrimidine-pyrimidone photoproducts are frequently found in skin cancer and its precursors. Therefore, removing these lesions is essential for the prevention of skin cancer. As UV-induced DNA damages are repaired by nucleotide excision repair (NER), the interaction of 1,25VD3 with NER components can be important for skin cancer transformation. Several studies show that 1,25VD3 protects DNA against damage induced by UV, but the exact mechanism of this protection is not completely clear. 1,25VD3 was also shown to affect cell cycle regulation and apoptosis in several signaling pathways, so it can be considered as a potential modulator of the cellular DNA damage response, which is crucial for mutagenesis and cancer transformation. 1,25VD3 was shown to affect DNA repair and potentially NER through decreasing nitrosylation of DNA repair enzymes by NO overproduction by UV, but other mechanisms of the interaction between 1,25VD3 and NER machinery also are suggested. Therefore, the array of NER gene functioning could be analyzed and an appropriate amount of 1.25VD3 could be recommended to decrease UV-induced DNA damage important for skin cancer transformation.

## 1. Melanoma and Non-Melanoma Skin Cancers

Skin cancer belongs to the most common human malignancies, with the highest rate of incidence in Caucasians [1]. In general, there are two distinct classes of skin cancer—cutaneous melanomas (CM) and non-melanomas. Non-melanoma skin cancers (NMSCs) can be categorized into two types, depending on their origin—basal cell carcinomas (BCCs), and squamous cell carcinomas (SCCs) [1]. Non-melanomas are much more frequent than melanomas, but they have better prognosis [1]. Both, basal and squamous cell carcinomas, originate from epidermal keratinocytes and both appear mainly in skin areas exposed to sunlight [2,3]. There is an increasing, global tendency in the incidence of all types of skin cancer. It is estimated that every year up to three million new cases of non-melanoma skin cancer cases occur worldwide [1,3]. The mortality of NMSC is higher for both, women and men, in Southern Europe, while lower in Scandinavia [4]. Basal cell carcinomas accounts for the majority of NMSC cases (about 80%–85%) and has a low rate of metastasis to other organs, while 15%–20% of NMSC are SSCs with a higher tendency to metastasize and higher mortality than BCCs [3,5,6]. SSCs in advanced stage can metastasize *via* lymphatic or hematogenous spread, and its metastatic rate varies between 3% and 10% depending on tumor location [7].

Malignant melanoma originates from epidermal melanocytes and can occur in any tissue, which contains such cells [7]. Its incidence is much lower than NMSCs, but it is highly prone to invade other tissues and has the highest mortality rate among skin cancers [1,2]. More than 130,000 new cases of melanoma are registered worldwide each year and incidence rate is considerably higher in Caucasians than African Americans [3]. As it is estimated by WHO each year about 65,000 people die from melanoma worldwide. A higher mortality rate and a poorer prognosis are registered for men [8].

The pathogenesis of skin cancers is complex and includes multiple mechanisms. Nevertheless, ultraviolet (UV) radiation is the major risk factor for both, melanoma and non-melanoma skin cancers [3]. UV light acts on skin cells in multiple ways. It predominantly causes oxidative stress, immunosuppression, inflammatory response and finally DNA damage [7,9]. It is estimated that protection from sunlight during childhood and adolescence may reduce the risk of NMSC by 78% [7,10]. It is also estimated that about 65%–90% of all melanoma cases are associated with UV light exposure [11]. UV radiation can diminish the functions of immune system by reducing cytokine production [12].

In general, skin cancer risk, as the risk of many other cancers, depends on the interaction between environmental, phenotypic and genetic factors. While UV light exposure is the leading environmental risk factor for all skin cancers, the phenotype predisposing for cutaneous melanoma is pale white skin, red or blond hair and blue eyes [13]. Genetic factors seem to be also important, since 10% of melanomas occurs in families with at least two melanoma cases among close relatives [14]. A number of genetic loci associated with skin cancer were identified, containing high- and low-penetrance mutations and high prevalence polymorphic variations [15]. About 30%–40% of familial melanoma cases is associated with inherited mutations in two genes—*CDKN2A* and *CDK4*, which are critical for cell cycle regulation [16]. Another gene with melanoma-related inherited mutation is BRCA-associated protein 1 (BAP1) [17]. Many polymorphic variants were associated with melanoma, including those located in melanocortin 1 receptor gene (*MC1R*), which encodes a receptor protein involved in melanogenesis [15,18].

## 2. Vitamin D and its Role in Physiology and Pathology

**Figure 1:**
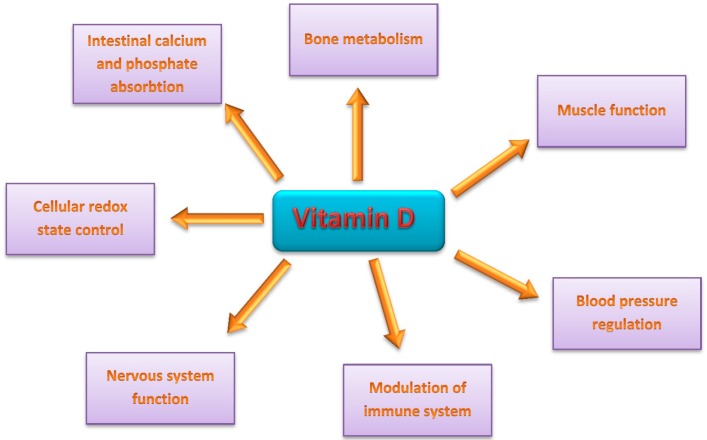
Multiple functions of vitamin D.

Vitamin D is essential for multiple physiological processes in the organism (Figure 1).

**Figure 2:**
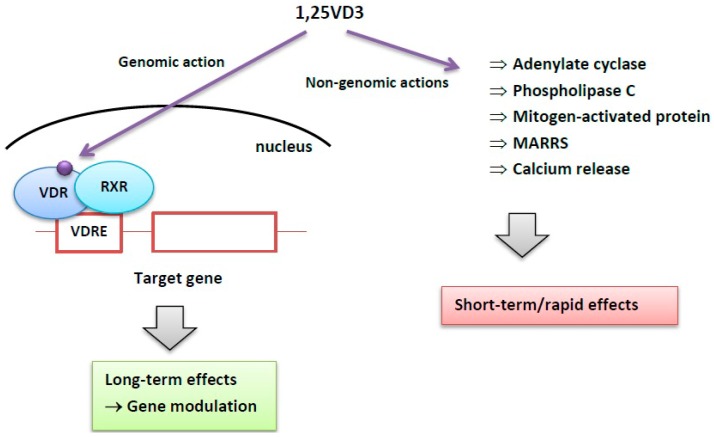
Genomic and non-genomic actions of vitamin D3. 1,25VD3 (1,25-dihydroxyvitamin D3)—active form of vitamin D; VDR, vitamin D receptor; RXR, retinoid X receptor; VDRE, vitamin D response element; MARRS, membrane-associated rapid-response steroid binding protein.

1,25-Dihydroxyvitamin D3 (1,25VD3) is an active, hormonal metabolite of vitamin D3 produced from its precursor, which comes from the diet or is synthesized from 7-dehydrocholesterol in the skin in a non-enzymatic reaction depending on UV energy [19]. 7-Dehydrocholesterol, present in both, epidermis and dermis, absorbs UVB (290–320 nm) light and changes into previtamin D3, which is followed by its thermal-dependent isomerization to cholecalciferol [20]. It is then transported to the liver and undergoes hydroxylation to 25-hydroxyvitamin D3 (25D), which is the major circulating form of vitamin D3 [19]. This hydroxylation is carried out by four cytochrome P-450 enzymes, three microsomal—CYP2R1, CYP2J2 and CYP3A4, and one mitochondrial, CYP27A1 [20,21]. Finally, it undergoes 1α-hydroxylation to form the active, hormonal form, 1,25VD3, which can be next transported to sites of its action bound to plasma vitamin D binding protein (DBP) [19] (Figure 2). The 1α-hydroxylation reaction takes place in kidney with the CYP27B1 hydroxylase responsible for this process [21], although it can also occur in other cell systems expressing CYP27B1, including muscles, pancreas, colon and cells of the immune system [20]. The activity of CYP27B1 is upregulated through parathyroid hormone (PTH) in response to low blood calcium level, while 1,25VD3 itself is a negative feedback factor for both PTH release and CYP27B1 activity [20,21].

Vitamin D plays a role in many physiological processes and its appropriate body level is essential. Severe deficiency of vitamin D leads to rickets, as vitamin D is key for the regulation of intestinal calcium and phosphate absorption, and thus for normal bone metabolism [22]. Proper vitamin D status is also important for muscle function (reviewed in [23]). Moreover, some results suggest that vitamin D can reduce pancreatic beta-cell destruction and in this way it can prevent autoimmune-based diabetes incidents [24]. In the cardiovascular system, vitamin D reduces systolic and diastolic blood pressure in hypertensive individuals and high vitamin D status is associated with 50% lower cardiovascular mortality risk [24,25]. It plays also a role in the modulation of the immune system, and low levels of vitamin D leads to disturbed immune functions and a high rate of infectious diseases [26]. Vitamin D may be also important for adequate nervous system function. Multiple sclerosis (MS) incidence was also found to correlate with vitamin D status. High vitamin D level was associated with lower MS incidence and low status of vitamin D correlated with a higher incidence of this disease [24,27]. In addition, it was recently reported that vitamin D played a role in the regulation of major histocompatibility complex (MHC) class II allele which is linked to MS [28].

Vitamin D plays a role in many signaling pathways, including those involved in control of the cellular redox state. It controls the expression of the Nrf2 (nuclear factor (erythroid-derived 2)-like 2) transcription factor, which activates many genes encoding antioxidant enzymes [29]. On the other hand, Nrf2 increases the expression of two genes—*FOS* and *JUN*—which in turn can induce the expression of genes important for vitamin D metabolism [30].

The mechanism of action by which the active form of vitamin D3 plays its roles splits into two main pathways—genomic and non-genomic (Figure 2).

The genomic action involves VDR—vitamin D receptor belonging to the nuclear receptors superfamily. VDR, while activated by 1,25VD3, dimerizes with the retinoid X receptor—RXR and this complex is ready for binding to vitamin D-responsive elements (VDREs) and in this way it can modulate the transcription of target genes [31]. At least 37 cell types express VDR and it was shown that 229 human genes contain VDRE sites, which suggest a multimodal role of vitamin D in the human organism [32,33]. Vitamin D regulates its target genes also through epigenetic mechanisms—it can regulate the methylation state of these genes and silence them [34,35].

In the non-genomic pathway vitamin D acts through membrane-bound receptors and second messengers, triggering a series of transduction systems. In many cell types, as keratinocytes, enterocytes, muscle cells or osteoblasts, 1,25VD3 activates cellular calcium influx and its intracellular release, modulates adenylate cyclase, phospholipase C or mitogen-activated protein (MAP) [36]. Some non-genomic actions of 1,25VD3 occur through membrane-anchored vitamin D receptors, as 1,25-dihydroxyvitamin D3-membrane associated rapid response steroid binding (MARRS) [36]. MARRS is located in the lipid rafts within membranes and can activate different kinases, phosphatases and ion channels [37].

The regulation of VDR gene expression occurs in a multi-level fashion and comprises environment influences, as well as genetic and epigenetic mechanisms [38]. Environmental factors, including diet, sun exposure, pollution and infections, regulate VDR mostly through modulating vitamin D3 level [38]. On the genomic level, the VDR gene is under the control of four promoters, which are tissue-specific and multiple alternative isoforms can be generated from them.

## 3. Vitamin D in Cancer

Emerging evidence suggests a role of vitamin D in cancer transformation. A number of epidemiological studies show the link between vitamin D status and different types of cancers. First suggestions came from an observation that a higher sunlight exposure was correlated with a reduced colon cancer mortality and a reduced risk of prostate cancer [39]. It was concluded that since sunlight exposure of skin was essential for vitamin D biosynthesis, higher levels of vitamin D played a role in the reduction of the risk of those cancers. Several further observations also confirmed this hypothesis, showing a protective effect of vitamin D in a large group of different cancer types, progression and overall mortality [40]. Interestingly, since UV-exposure is the main risk factor for melanoma, associated with UV-exposure vitamin D synthesis may also serve as a protective factor [41,42]. Although the incidence of melanoma is higher in southern countries, at the same time melanoma prognosis is also better for populations from these countries [42]. In addition, it was shown that low vitamin D status was associated with a progression of melanoma [41]. In a number of studies in different populations a significant association was found between serum vitamin D status and prostate cancer risk and its aggressiveness [43,44,45]. Probably, the most convincing results on the association of vitamin D with malignant transformation come from colorectal cancer. It was shown that the risk of colorectal cancer inversely correlated with serum vitamin D concentration and its dietary intake [40,46,47,48,49]. A number of studies suggest also a link between breast cancer and vitamin D, although some data are inconsistent. Several reports show a straight reduced risk of breast cancer incidence in patients with higher vitamin D concentrations or consumption [50,51,52]. However, other studies showed conflicting results of a U-shaped relationship or no association at all between vitamin D status/intake and breast cancer risk [52,53,54]. Another evidence comes from studies on animal models showing that deletions in the VDR gene were associated with an increased cancer risk [55,56], and that injection of chemical analogues of vitamin D led to a reduction in tumor incidence and size [55,56,57,58,59,60].

**Figure 3:**
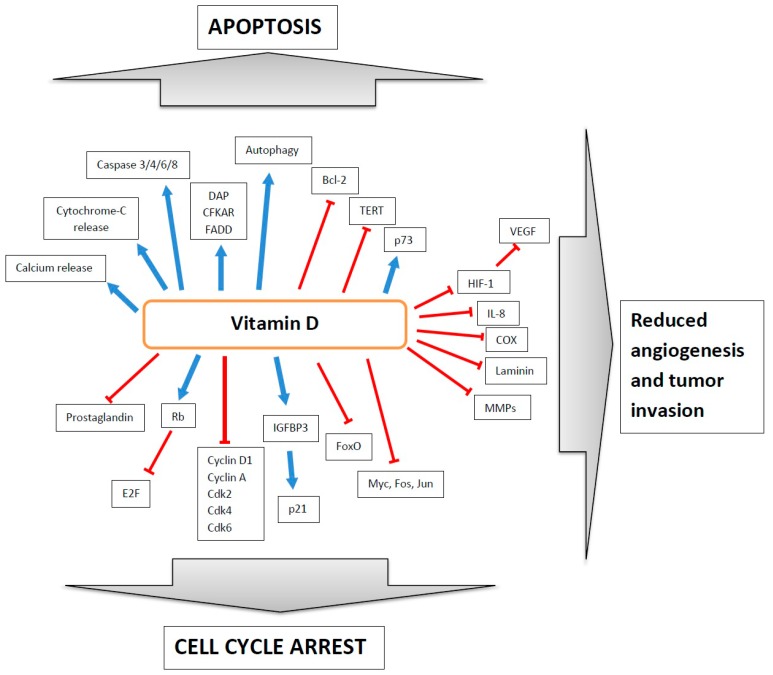
Anticancer action of vitamin D. Sharp blue arrows—stimulation, blunt red arrows—inhibition. DAP—death-associated protein-3; CFKAR—caspase 8 apoptosis-related cysteine peptidase; FADD—Fas-associated death domain; Bcl-2—B-cell lymphoma 2 (antiapoptotic factor); TERT—telomerase reverse transcriptase; HIF1—hypoxia induced factor 1; VEGF—vascular endothelial growth factor; IL-8—interleukin 8; COX—cyclooxygenase; MMPs—matrix metalloproteinases; Rb—retinoblastoma protein (tumor suppressor); E2F—transcription factor, regulator of cell cycle; Cdk2/4/6—Cyclin-dependent kinases; IGFBP3—insulin-like growth factor binding protein-3; FoxO—Forkhead box O transcription factor; Myc/Fos/Jun—oncogenes involved in cell cycle and apoptosis regulation.

Vitamin D may exert its anticancer roles in a number of ways, but its anti-proliferative potential seems to be best documented among them (Figure 3).

Vitamin D was shown to exert a dose-dependent inhibitory effect on melanoma cell growth [61]. It was confirmed in a number of other cancer cells including breast cancer, colon cancer or prostate cancer cells [62,63,64]. Other results suggested that vitamin D could have antiproliferative properties for both normal and cancer cells expressing VDR and normal cells, and high doses of vitamin D3 led to G0/G1 cell cycle arrest [65]. Active vitamin D was found to influence the phosphorylation state of retinoblastoma protein (Rb), which is an important regulator of the cell cycle, directing progression from G1 to S [65]. After dephosphorylation of Rb stimulated by vitamin D, it binds to the E2F transcription factor, which is important for the cell cycle [65]. Another mechanism of vitamin D action in cancer can be its inhibitory effect on the expression of cyclin D1E and cyclin A, and kinases CDK2, 4 and 6 [65]. Moreover, vitamin D decreases the activity of prostaglandins, which stimulate cell growth and promote its degradation [66]. In prostate cancer cell line vitamin D led to upregulation of insulin-like growth factor binding protein-3 (IGFBP3), which can inhibit cell cycle through upregulation of p21 expression [67,68]. Other cell cycle regulators affected by vitamin D are Forkhead box O transcription factors (FoxO). Vitamin D promotes the interaction between FoxOs and its regulators, Sirt1 and protein phosphatase 1, leading to dephosphorylation of FoxOs and its suppressive action on genes involved in proliferation control [69]. Finally, vitamin D can also affect the expression of other genes involved in proliferation control, including *Myc*, *Fos* and *Jun* [70].

Apart from its antiproliferative function, vitamin D can also promote apoptosis, as it was shown on a number of cancer cell lines. Vitamin D induced the expression of the p73 protein, which plays a role in apoptosis induction in cancer cells. On the other hand, the suppression of p73 led to a decrease in the pro-apoptotic action of vitamin D [71]. As it was shown on prostate and breast cancer cell line, vitamin D induced apoptosis of cancer cells through the disruption of mitochondrial function, releasing cytochrome C and generating reactive oxygen species (ROS) [39]. It seems that vitamin D acts through the inhibition of the anti-apoptotic factor Bcl-2 [72]. Other pro-apoptotic genes, which are induced by vitamin D are *DAP* (death-associated protein-3), *CFKAR* (caspase 8 apoptosis-related cysteine peptidase), and *FADD* (Fas-associated death domain) [37]. In addition, vitamin D induced caspases 3, 4, 6 and 8 [37,73]. Vitamin D also sensitizes cancer cells to apoptosis stimulated by oxidative stress and cytokines [37]. It also triggers calcium release and influx thus promoting apoptosis through activation of calcium-dependent μ-calpain and calcium/calpain-dependent caspase 12 [37,74]. Another proposed mechanism engages telomerase reverse trancriptase (TERT). Vitamin D in ovarian cancer cells can induce apoptosis by inhibiting TERT activity and subsequently inducing apoptosis through telomere shortening [75]. Finally, vitamin D stimulates autophagy in a number of cancer cell lines through inhibition of the anti-autophagic gene *mTOR* and stimulation of the pro-autophagic gene *beclin-1* [37,76].

A number of *in vitro* and *in vivo* studies show that vitamin D can inhibit angiogenesis in tumor formation. Vascular endothelial growth factor (VEGF) is the major pro-angiogenic element responsible for tumor vascularization. VEGF expression is upregulated in response to hypoxia through the action of HIF1α (hypoxia induced factor 1 α) [77]. Vitamin D was reported to reduce tumor vascularization by inhibition of endothelial cells proliferation [77]. In a hypoxic state vitamin D reduced VEGF expression, possibly through the inhibition of HIF-1 expression [78]. Moreover, increased expression of pro-angiogenic factors—HIF-1, VEGF, angiopoietin and platelet-derived growth factor (PDGF)—was observed in a VDR knockout mouse model [79]. In addition, vitamin D suppressed the expression of the proangiogenic IL-8 factor independently of nuclear factor kappa-light-chain-enhancer of activated B-cells (NFκ-B) [80]. Another indirect mechanism can be dependent on cyclooxygenase-2 (COX-2), which can promote angiogenesis and tumor progression. Vitamin D3 may also inhibit angiogenesis through the suppression of *COX* gene expression [81]. Additionally to antiangiogenic action, vitamin D may also reduce the invasion of tumor cells, possibly thanks to downregulation of the matrix protein laminin and its receptors [82,83]. Vitamin D can also indirectly inhibit the activity of key factors for metastasis—matrix metalloproteinases (MMPs) and cathepsins, through induction of their inhibitors [84]. It was shown that 1,25VD3 inhibited the conversion of the inactive form of MMP-9 stimulated by interleukin 1β (IL-1β) reversing an inhibitory effect of IL-1β on the production of MMPs tissue inhibitors, TIMP-1 (tissue inhibitor of matrix metalloproteinases-1) and TIMP-2 [85].

Vitamin D was shown to interact with a number of oncogenes by a direct inhibition of their expression and these interactions depended on tumor type. It was shown that vitamin D significantly reduced the expression of the *c-myc* oncogene in breast and ovarian cancer cell lines [86,87]. Similar effects were also observed in prostate cancer cells [88]. Vitamin D reduced also the expression of the *c-fos* oncogene. Importantly, both, *c-myc* and *c-fos* genes contain VDREs in their promoters [89,90,91]. In breast and cervical cancer cell lines vitamin D inhibited the expression of the *KCNH1* oncogene, encoding voltage-gated channel Eag1, and that inhibition was accompanied with a reduction in cell proliferation and decreased tumor growth [92,93].

Finally, VDR was identified as a tumor suppressor in the skin, with three predominant mechanisms underlying its function: inhibition of proliferation and induction of dedifferentiation, immune control and involvement in DNA damage response (DDR) [94].

## 4. DNA Damage Induced by UV Radiation and Its Role in Skin Carcinogenesis

UV radiation can be classified into three wave-length ranges: UVA (320–400 nm), UVB (290–320 nm) and UVC (100–290 nm). Solar UV radiation reaching the earth consists mainly of UVA and UVB, because radiation below 300 nm weakly penetrates the ozone layer in the atmosphere. Carcinogenic effects of UV light are attributed to its DNA-damaging properties (reviewed in [95]).

Two main DNA damage types induced by UV radiation, cyclobutane pyrimidine dimer (CPD) and (6-4) pyrimidine-pyrimidone photoproduct (6-4PP), result from a direct absorption of the radiation by DNA and are highly specific to UV. Several other photoproducts can be induced by UV radiation as well as other DNA damages, including DNA-protein cross-links and strand breaks (reviewed in [96]). CPD and 6-4PP are bulky adducts, which, if not repaired, can interfere with the progression of DNA replication, blocking the movement of replicative DNA polymerase, which can be replaced by translesion synthesis (TLS) DNA polymerases, which are error-prone and can induce mutations. These mutations can play a direct role in skin cancer transformation if they occur in oncogenes, including the *ras* gene, tumor suppressor genes, including the *TP53* and *ptc* genes or DNA maintenance genes. However, it should be taken into account, that some TLS polymerases can be error-free, depending on the lesion context [97]. In addition, CPDs can block the binding of transcription factors, if they occur in the promoter sequences and inhibit transcription elongation [98,99]. UV-induced DNA damage can also induce replication fork collapse [100]. All these scenarios increase genomic instability of UV-irradiated cells, which can be a prerequisite for cancer transformation. Other carcinogenic effects of UV-induced mutations can include altering the function of genes involved in the transduction of signals involved in the process of cancer transformation [101].

The DNA-damaging effect induced by UV can also result from the production of ROS and this mechanism seems to be of a particular importance in the case of UVA as this UV component is mostly absorbed by chromophores other than DNA, which can change the cellular redox equilibrium leading to ROS production [102].

UV radiation can also contribute to skin carcinogenesis by mechanisms not directly related to DNA damage. An example can be the interaction between UV and epidermal growth factor receptor (EGFR), which in normal conditions is in an inactive state due to the interaction with protein tyrosine phosphatase κ (PTPRK) [103]. UV radiation can block the activity of PTPRK enabling phosphorylation of EGFR, transforming it in its active form, which can play an important role in skin carcinogenesis. This is also an example illustrating a non-direct effect of CPD and 6-4PP-related mutations, if they occur in the PTPRK gene and inactivate its product.

UV radiation-mediated immunomodulation is an important mechanism of skin carcinogenesis [104]. In humans, CPD and 6-4PP are removed by nucleotide excision repair (NER) and defects in this DNA repair system can result in severe disorders, including *xeroderma pigmentosum* (XP), characterized by an increasing sensitivity to sunlight, resulting in squamous cell carcinoma, basal cell carcinoma and melanoma skin cancer (reviewed in [105]). *Xeroderma pigmentosum* was successfully targeted by gene therapy (reviewed in [106]). Patients with malfunctioning NER show up to 10^4^ increase in skin cancer risk and the onset of this disease is at apparent lower age than typical for general population [107]. Some reports suggest the involvement of mismatch repair (MMR) in processing of UV-induced DNA damage, in particular damage resulting from replication errors on repeats-containing template, but the association of MMR with skin carcinogenesis is still controversial [108]. Therefore, efficient functioning of NER is crucial for preventing UV radiation-induced skin cancer transformation.

In general, activation of signaling pathways can play an important role in skin carcinogenesis. It was shown that solar radiation, containing UVA and UVB, significantly affected the p38 MAPK (mitogen-activated protein kinase)-related signaling pathways [109]. These pathways can be involved in DDR, as they can participate in apoptosis and autophagy [110,111]. Apoptosis, autophagy and senescence can be regulated also by the activation of the AKT/mTOR (serine/threonine protein kinase B/mammalian target of rapamycin) signaling pathway in response to UV radiation (reviewed in [112]). This activation can be important for skin carcinogenesis by modulation of DDR in reply to UV-induced DNA damage by inhibition of apoptosis and induction of the transition of arrested cell cycle as well as inhibition of autophagy. These actions are counteracted by p53, which is activated in response to UV-induced DNA damage, so the interplay between p53 and AKT/mTOR can be important for UV-induced skin carcinogenesis. Other signaling pathways induced by UV, including Jun N-terminal kinase (JNK) and nuclear factor kappa-light-chain-enhancer of activated B cells (NF-κB), can play a role in skin cancer [113]. Therefore, several UV-activated signaling pathways should be taken into account in considering mutual relationships between skin carcinogenesis and NER in the presence of vitamin D3, as at least some of them can be associated with NER through their involvement in DDR.

## 5. Nucleotide Excision Repair—The Most Versatile DNA Repair System

Nucleotide excision repair acts on a broad spectrum of substrates, which cause a distortion in the DNA helix and introduce its chemical modification [114]. These are mainly bulky covalent adducts with the involvement of the DNA bases affected by UV and ionizing radiation, chemicals, including many anticancer drugs and environmental pollutants and some endogenous compounds, including reactive oxygen and nitrogen species. Other NER substrates are apurynic/apyrimidinic sites (AP sites). NER can also cooperate with homologous recombination repair in removing DNA interstrand crosslinks, which are one of the most, if not the most, serious DNA damage [115]. An undamaged DNA strand opposite to the damage is necessary to serve as a template for DNA repair synthesis in NER.

NER is often categorized as global genome repair and transcription-coupled repair. In light of pervasive transcription of the human genome, the latter is of a special significance, especially that its defects are associated with serious human disease (see below).

**Figure 4:**
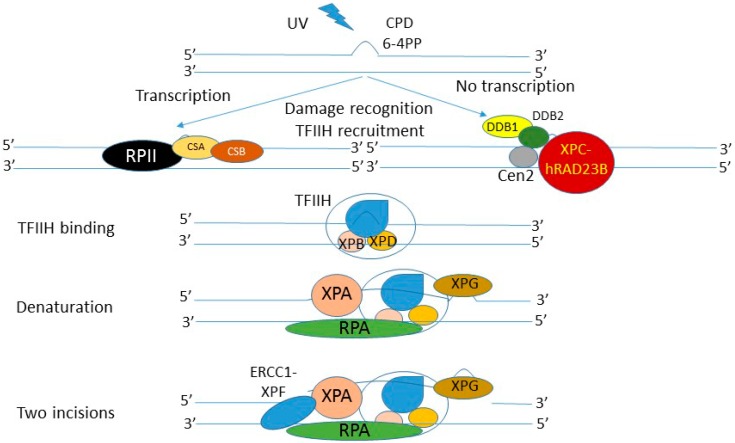
Simplified scheme of nucleotide excision repair of cyclobutane pyrimidine dimer (CPD) and pyrimidine-pyrimidone (6-4) photoproduct (6-4PP) induced by UV radiation. Several other proteins, not presented here, can be involved in this process. Repair synthesis, which is normally led by replicative DNA polymerase is not presented. RPII, RNA polymerase II; XPB,D,A,G,C: *xeroderma pigmentosum* complementation group B,D,A,G,C; ERCC1-XPF: excision repair cross-complementation group 1-*xeroderma pigmentosum* complementation group F; DDB1: DNA damage binding protein 1; Cen2: Centrin 2; hRAD23B: protein involved in DNA damage recognition.

NER starts with damage recognition, in which the XPC (*xeroderma pigmentosum* complementation group C) protein plays the major role (Figure 4). It acts in a complex with hRAD23B, but it is able to bind damaged DNA alone with no relation to NER [116]. For some DNA damage, including CPDs and 6-4PP, the process of lesion recognition is supported by DNA damage-binding protein 1 and 2 (DDB1 and 2) and it was suggested that the DDBs along with other proteins, mediated targeting of the XPC-hRAD23B complex to the CPD/6-4PP site [117]. When CPD occurs in an actively transcribed region of a genome, its recognition is mediated by two proteins of the Cockayne syndrome group: CSA and CSB, which release arrested RNA polymerase II (reviewed in [118]). The XPC-hRAD23 complex with DDBs can be assisted by another protein, centrin-2 (Cen2), which can play a role in the regulation of the transcription factor TFIIH (general transcription factor for RNA polymerase II H) recruitment to the site of damage [119]. TFIIH, a multimeric protein complex, conducts two critical NER steps: damage verification and unwinding the DNA helix around the damage. The latter is done due to two helicase activities of TFIIH originating from its two subunits, XPB (*xeroderma pigmentosum* complementation group B) and XPD (*xeroderma pigmentosum* complementation group D). Other proteins, XPA (*xeroderma pigmentosum* complementation group A) and RPA (replication protein A), determine the site of cleavage and strand specificity and recruit XPG (*xeroderma pigmentosum* complementation group G, ERCC5 (excision repair cross-complementation group 5)), which incises DNA at the 3′ side of the damage, whereas incision on the other side is made by the ERCC1-XPF (excision repair cross-complementation group 1-*xeroderma pigmentosum* complementation group F) complex. This results in excision of the DNA fragment containing the damage, which is replaced by a new one in the process of repair synthesis led by replicative DNA polymerase δ/ε, assisted by proliferating cell nuclear antigen (PCNA) and RPA as well as replication factor C (RFC) [120].

Because NER removes a fragment of DNA containing damage, this can lead to the removal of some information, other than DNA sequence, contained in this fragment. An important example of this effect is lack of methylation signals in the sequences adjacent to damage site just prior and after the process of DNA replication [121]. If this occurs in a gene region, it can interfere with the regulation of its expression.

Human NER, as a cellular response to UV radiation-induced DNA damage, operates within chromatin, which must be remodeled to enable effective DNA repair. However, structural changes in chromatin in response to DNA damage are necessary for all DNA repair pathways, so they will not be considered in this review, despite that some of them can be specific to UV radiation (reviewed in [122]).

## 6. Vitamin D and DNA Damage/Repair Induced by UV Radiation

Several studies showed that 1,25VD3 protected against DNA damage induced by UV. De Haes *et al.,* demonstrated that 1,25VD3 and its low-calcemic analogues decreased the level of CPDs in human keratinocytes exposed to UV [123]. The authors considered antiproliferative potential of 1,25VD3 as the major factor involved in the mechanism of that protection against formation of pyrimidine dimers. It was to be associated with the induction of growth inhibition due to the cell cycle arrest, in which DNA is in a more packaged form and less accessible to DNA-damaging factors. The protection of skin against UV-induced DNA damage was reported for several substances, including retinyl esters, synthetic UV filters, polyphenols extracted from green tea, genistein and others, which could absorb UV radiation [124,125,126,127]. However, this mechanism could not be taken into account in considering the protective action of 1,25VD3 against UV-induced DNA damage due to small size of the vitamin and lack of UV-absorbing residues.

When the extent of DNA damage exceeds the repair capacity of the cell, the cell cycle can be arrested, first of all at the G1/S and G2/M checkpoints and the cell could have some more time to repair the damage. However, if this prolonged time is not sufficient, the cells can enter a programmed death pathway, usually apoptosis, to avoid cancer transformation. Therefore, it is crucial that the cells with highly damaged DNA by UV, would be directed to apoptosis. However, 1,25VD3 prevents not only UV-induced DNA damage, but UV-induced apoptosis as well [128]. Therefore, there is somehow a puzzled situation—UV radiation induces DNA damage, but stimulates keratinocytes to release vitamin D-precursor, which is then metabolized to 1,25VD3, which protects keratinocytes against UV-induced DNA damage. However, when the extent of DNA damage is too high, 1,25VD3 prevents apoptosis, which can support UV-induced skin cancer transformation, but such mechanism seems to be unlikely and the protective action of VD3 against UV-induced apoptosis results rather from decreasing the extent of DNA damage below the threshold needed to switch on apoptotic machinery. Therefore, the effect of vitamin D3 on apoptosis can be important for UV-induced skin carcinogenesis. Some general aspects of this issue were addressed earlier in this manuscript.

Vitamin D3 was shown to activate pro-survival pathways in skin cells: the ERK (MEK/extracellular signal regulated kinase) pathway and the PI-3K/AKT (phosphatidylinositol 3-kinase/AKT) pathway in response to apoptotic stimuli [129,130,131]. In addition, 1,25VD3 was shown to modulate the expression of Bcl-2-family proteins, including Bcl-2, Bax and Bad, and the relationship between levels of their expression suggests its anti-apoptotic action. However, it was shown that 1,25VD3 decreased the expression of the anti-apoptotic Bcl-xl (B-cell lymphoma-extra large) protein in psoriatic lesions in cultured human keratinocytes [132]. Therefore, anti- or pro-apoptotic action of 1,25VD3 can be dependent on the physiological/pathological conditions in a cell, in particular on the development of cancer transformation, which is associated with the activation of different signaling pathways, which can be differently modulated by 1,25VD3. For example, Wnt/β-catenin signaling is involved in “phenotype switching” between highly proliferative and highly invasive phenotypes in melanoma (reviewed in [133]) and 1,25VD3 was reported to modulate this signaling pathways in skin cells, although this effect resulted mainly from the interaction of the vitamin with its receptor, VDR [37]. Therefore, further studies are needed to determine the role of 1,25VD3 in apoptosis of skin cancer cells.

It was shown that 1,25VD3 protected human keratinocytes from UV-induced DNA damage in the form of thymine dimers, strand breaks and oxidative DNA damage by decreasing the level of reactive nitrogen species (RNS) [134]. It was supported by the observation that RNS can induce DNA damage in various forms, including strand breaks and base modifications (reviewed in [135]). However, a question arises whether this effect can be associated with the influence of RNS on the process of DNA repair. Obviously, RNS can attack DNA repair proteins leading to inhibition of their activity and decreasing the efficacy of DNA repair, but this effect is non-specific. As peroxynitrite (ONOO^−^) can be considered as a major RNS/ROS and it is a NO derivative, overproduction of nitric oxide can result in an excess of peroxynitrite. It was demonstrated that an elevated NO synthesis was associated with DNA repair inhibition by nitrosylation of DNA repair enzymes [136]. It was also shown that an excess of NO could affect the excision and ligation steps in NER of pyrimidine dimers [137]. Several other works showed the modulation of DNA repair by nitric oxide (reviewed in [138]). Therefore, 1,25VD3 can influence DNA repair, including NER, by decreasing the production of nitric oxide. 1,25VD3 not only reduced the extent of nitric oxide products, but also increased the expression of p53, suggesting its involvement in DDR, including DNA repair [139].

**Figure 5:**
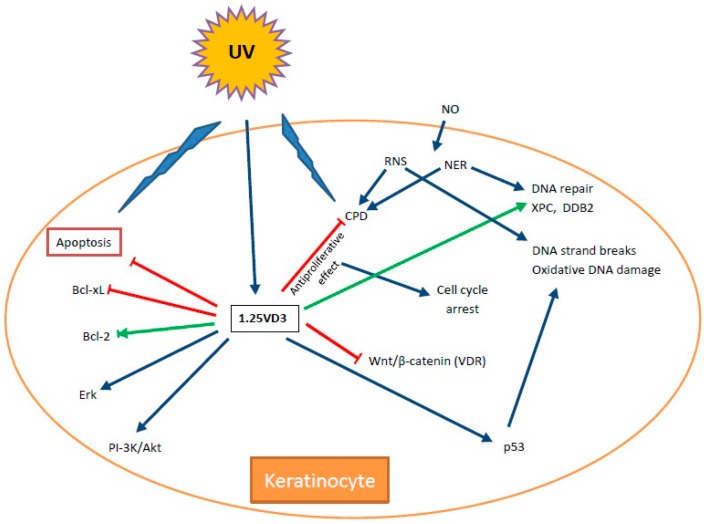
Some pathways and interactions important for the involvement of vitamin D3 in DNA damage response (DDR). UV radiation can induce DNA damage, which here is represented by the cyclobutane pyrimidine dimer (CPD). It can also directly induce apoptosis and stimulate the production of 1,2-dihydroxywitamin D3, an active metabolite of vitamin D3, which can, in turn, affect various aspects of DDR as described in text. Green sharp arrows—activation, red blunt arrows—inhibition, dark blue sharp arrows—general influence. NO: nitric oxide; RNS: reactive nitrogen species; NER: nucleotide excision repair.

In UVB-irradiated mice, the removal of CPD was significantly faster in epidermal keratinocytes of wild-type animals than in vitamin D receptor double mutants (*VDR^−/−^*) [140]. In similar research, but performed *in vitro*, no difference in the initial level of CPDs and 6-4PPs was observed between wild-type keratinocytes and their *VDR-*negative counterparts, as evaluated by standard immunoblot assay [141]. However, after 48 h, both cell types were deficient in CPDs repair and displayed similar efficacy in removing 6-4PPs. The authors interpreted obtained results as an example of so called rodent repair paradox (repairadox)—rodent cells show a decreased rate of removing some kinds of UV-induced DNA damage than human cells, but they display a similar ratio of survival [142,143]. Therefore, VDR can play a role in NER processing of UV-induced DNA damage. Moreover, this role seems to result in stimulating this kind of DNA repair. Consequently, VDR might be induced by UV and stimulate NER. This hypothesis was positively verified by several studies showing induction of VDR by UVB [144,145,146]. However, another study reported VDR downregulation by UVB [147]. In general, this seems to be a complex problem involving the balance between physiology and carcinogenesis, as VDR determines the response of keratinocytes to the hormonally active form of vitamin D3, 1,25VD3, which, in turn, can change their differentiation and proliferation, which are essential in cancer transformation [148] (Figure 5).

All these data suggest a protective action of 1,25VD3, resulting mainly from the involvement of VDR, against DNA damage induced by UV radiation [129,149]. As the damage is represented mostly by the UV photoproducts removed by NER, it is tempting to speculate that the vitamin can stimulate NER. As mentioned above, the TFIIH transcription factor plays an important role in NER and mutations in its XPD component are associated with XP and trichothiodystrophy, DNA repair disorders [150]. Moreover, some XP-related symptoms were associated with defects in the vitamin D pathway. In an elegant work, Drane and coworkers showed an inhibition of transactivation by VDR in XPD-deficient cells, mainly by the preventing its binding to the promoter of some responsive genes [151]. This transactivation defect was associated with defective Ets1, a transcription factor, which cannot be phosphorylated by TFIIH in XPD-deficient cells, as phosphorylated Ets1 supports binding of VDR to its responsive elements. However, as TFIIH plays a dual role in both transcription and DNA repair, the question whether this mechanism is DNA repair-dependent or -independent remains open. An increased expression of two important NER genes, *XPC* and *DDB2*, was observed in primary human keratinocytes upon 1,25VD3 treatment [152].

Some data suggest a functional involvement of NER factors in transcription beyond the canonical NER pathway (reviewed in [153]). It seems important to study a potential link of such action and vitamin D3 metabolism.

## 7. Conclusions and Perspectives

DNA nucleotide excision repair (NER) system plays an important role in skin carcinogenesis as it removes pre-carcinogenic DNA lesions induced by UV radiation. However, UV is important for the production of vitamin D3, indispensable in bone development. An emerging body of evidence suggests that there is a thin line between physiological and pro-carcinogenic effects of VD3. The width of this line depends on the influence of the vitamin on the pro-carcinogenic features of UV action. These features are expressed by the extent of DNA damage induced by the radiation, primarily proceeded by NER. Therefore, it is important to determine whether VD3 can modulate NER. In this review we tried to explore this issue, however it seem that no definite conclusion can be drawn at present, because some studies have shown conflicting and equivocal results. Although there is a general agreement that 1,25VD3 could protect the cell against DNA lesions induced by UV, no clear evidence on the mechanism underlying this effect is provided. Therefore, determining this mechanism is an important task.

Several pathways can be considered in the protective mechanism of 1,25VD3 against DNA damage induced by UV. However, some of them can be seen as a two-edged sword. The involvement of the vitamin in pathways related to apoptosis can have contrasting consequences. Activating pro-survival pathways by the vitamin can lead to an expansion of subpopulation of cells with UV-damaged DNA, but some reports suggest its pro-apoptotic action. This is especially intriguing in the context of NER functioning, as apoptosis can be turned on when the cell has no chance to complete the repair of the existing extent of DNA damage in a reasonable time. The assessment of this chance depends on the level and activity of NER proteins, predominantly involved in repairing of UV-induced DNA damage. Therefore, details of possible interaction of 1,25VD3 with NER components would add important information on the mechanism of its protective action against DNA damage induced by UV. However, our knowledge of this issue is scarce. Moreover, it seems that the key role in this mechanism can be played by the vitamin D3 receptor, VDR, so that the research on the cross-talk between NER proteins, vitamin D3 and its receptor could bring significant results contributing to the determination of the relationship between pro-carcinogenic influence of UV and protective action of 1,25VD3.

Although there is very little information on the direct interaction between the NER machinery and vitamin D3, studies on this subject in the perspective of skin cancer prevention and therapy are justified. Effective NER can remove a substantial fraction of UV-induced photoproducts, decreasing the chance of cancer transformation in the skin. Therefore, as there are suggestions that in certain cases 1,25VD3 can stimulate NER, these cases should be determined in order to find the optimal conditions for NER action in the presence of VD3. This can be important for skin cancer prevention. Although the anticancer use of 1,25VD3 in general is limited to preventive therapy its potential as a predictor of occurrence and overall survival in cancer is also considered along with its receptor [154]. The problem of therapeutic use of vitamin D in skin cancer is complicated by the fact that UV is the main etiological factor of skin cancer, but about 90% of vitamin D needed for the human body should be formed by the action of UV in the skin. Therefore, protection against UV can result in 1,25VD3 deficiency leading to serious pathologies, including bone diseases, hypertension and cardiac diseases as well as several cancer types. Consequently, it is reasonable to assume that the current level of solar UV in certain locations, including Europe and North America is “physiological”. The problem of evaluation of the “adequateness” of UV intensity in other sites, like Australia or North Africa, is difficult as it can be linked with many aspects, including ethnicity and health care system.

Some reports show a potential of direct application of vitamin D in cancer treatment, including targeting cancer stem cells [155,156], but this is still controversial. Instead, vitamin D can play an important, but mainly auxiliary role in diagnosis, prognosis and prediction of many cancers and its monitoring can imply changes in chemotherapeutic regime. Nevertheless, the preventive role of vitamin D3 against cancer development seems to be its most important potential function in cancer therapy. However, epidemiologists anticipate, the influence of primary prevention on melanoma incidence rate in Europe is unlikely to be seen in the near future and instead an increase is expected [150]. Therefore, it is important to look for new therapeutic strategies in skin cancer.

In this review we have suggested that as the DNA nucleotide excision repair system plays an important role in skin carcinogenesis due to the removal of UV-induced carcinogenic DNA lesions, and that there is a thin line between physiology and pathology of vitamin D3 produced in skin with UV radiation, the vitamin can modulate NER and this can be exploited in skin cancer prevention. As we stated, it is likely that 1,25VD3 can stimulate NER, leading to more efficient removal of carcinogenic UV-induced photoproduct and other lesions, involved in skin cancer transformation. A topical application of vitamin D is a trivial preventive procedure, but its efficacy against skin cancer formation cannot be easily assessed, especially in the context of NER functioning. In the near future a skin cancer diagnostic/preventive procedure could look as follows: keratinocytes would be isolated from an individual and analyzed on the efficacy of NER with a functional assay, but not a kinetics of photoproduct removing, because their number can vary not only due to DNA repair, but other factors as well; then a dedicated microarray with NER genes is analyzed, indicating potential disturbances in the expression of essential NER components; finally, a dose of supplementation with vitamin D3 would be determined, taking into account individual relationship between UV sensitivity and NER efficacy.

Two final points can be made. Firstly, there is a mutual relationship between skin cancer, UV-exposure, vitamin D3 production and nucleotide excision repair functioning. This relationship should be studies in detail to determine physiological and pathological levels of exposure to UV and 1,25VD3 production in dependence on the efficacy of NER. Secondly, supplementation with 1,25VD3 could be considered as a strategy in skin cancer prevention in dependence with the individual’s NER capacity.

## Abbreviations

The following abbreviations are used in this manuscript (in order of appearance):

1,25VD3	1,25-Dihydroxyvitamin D3
VDR	Vitamin D receptor
NER	Nucleotide excision repair
CM	Cutaneous melanoma
BCC	Basal cell carcinoma
SCC	Squamous cell carcinoma
NMSC	Non-melanoma skin cancer
BAP1	BRCA-associated protein 1
CYP2R1, CYP2J2, CYP3A4, CYP27A1, CYP27B1	cytochrome P-450 enzymes
PTH	Parathyroid hormone
MS	Multiple sclerosis
MHC	Major histocompatibility complex
Nrf2	Nuclear factor (erythroid-derived 2)-like 2
FOS, JUN	Transcription factors
RXR	Retinoid X receptor
VDRE	Vitamin D response element
MARRS	Membrane-associated rapid-response steroid binding protein
DAP	Death-associated protein-3
CFKAR	Caspase 8 apoptosis-related cysteine peptidase
FADD	Fas-associated death domain
Bcl-2	B-cell lymphoma 2 antiapoptotic factor
TERT	Telomerase reverse trancriptase
HIF1	Hypoxia induced factor 1
VEGF	Vascular endothelial growth factor
IL-8	Interleukin 8
COX	Cyclooxygenase
MMP	Matrix metalloproteinase
Rb	Retinoblastoma protein
E2F	Transcription factor, regulator of cell cycle
Cdk2/4/6	Cyclin-dependent kinases
IGFBP3	Insulin-like growth factor binding protein-3
FoxO	Forkhead box O transcription factor
ROS	Reactive oxygen species
DAP	Death-associated protein-3
CFKAR	Caspase 8 apoptosis-related cysteine peptidase
FADD	Fas-associated death domain
mTOR, beclin-1	Proteins involved in autophagy
NFκ-B	Nuclear factor kappa-light-chain-enhancer of activated B-cells
COX-2	Cyclooxygenase-2
DDR	DNA damage response
CPD	Cyclobutane pyrimidine dimer
6-4PP	Pyrimidine-pyrimidone (6-4) photoproduct
TLS	Translesion synthesis
EFGR	Epidermal growth factor receptor
PTPRK	Protein tyrosine phosphatase kappa
MAPK	Mitogen-activated protein kinase
AKT/mTOR	Serine/threonine protein kinase B/mammalian target of rapamycin
XP	Xeroderma pigmentosum
XPA-G	Xeroderma pigmentosum complementation group A-GDDB1 and 2: DNA damage-binding protein 1 and 2
hRAD23B	Protein involved in damage recognition in nucleotide excision repair
Cen2	Centrin-2
RPA	Replication protein A
PCNA	Proliferating cell nuclear antigen
TFIIH	General transcription factor of RNA polymerase II H
CSA, CSB	Proteins of the Cockayne syndrome group: A and B
RFC	Replication factor C
ERK	MEK/extracellular signal regulated kinase pathway
PI-3K/AKT	phosphatidylinositol 3-kinase/Serine/threonine protein kinase B
Bcl-2, Bax and Bad	proteins of Bcl-2 family of anti-apoptotic proteins
Bcl-xL	B-cell lymphoma-extra large protein
RNS	Reactive nitrogen species
